# Appendage-Bearing *Sordariomycetes* from *Dipterocarpus alatus* Leaf Litter in Thailand

**DOI:** 10.3390/jof9060625

**Published:** 2023-05-29

**Authors:** Nethmini P. Samaradiwakara, Antonio Roberto Gomes de Farias, Danushka S. Tennakoon, Janith V. S. Aluthmuhandiram, Chitrabhanu S. Bhunjun, K. W. Thilini Chethana, Jaturong Kumla, Saisamorn Lumyong

**Affiliations:** 1 Department of Biology, Faculty of Science, Chiang Mai University, Chiang Mai 50200, Thailand; nethmips@gmail.com (N.P.S.); danushkasandaruwanatm@gmail.com (D.S.T.); jaturong.kumla@gmail.com (J.K.); 2 Research Center of Microbial Diversity and Sustainable Utilization, Faculty of Science, Chiang Mai University, Chiang Mai 50200, Thailand; 3 School of Science, Mae Fah Luang University, Chiang Rai 57100, Thailand; janithvishvakeerthi9@gmail.com (J.V.S.A.); avnishbhunjun@gmail.com (C.S.B.); kandawatte.thi@mfu.ac.th (K.W.T.C.); 4 Center of Excellence in Fungal Research, Mae Fah Luang University, Chiang Rai 57100, Thailand; antonio.gom@mfu.ac.th; 5 Beijing Key Laboratory of Environment Friendly Management on Fruit Diseases and Pests in North China, Institute of Plant and Environment Protection, Beijing Academy of Agriculture and Forestry Sciences, Beijing 100097, China; 6 Academy of Science, The Royal Society of Thailand, Bangkok 10300, Thailand

**Keywords:** *Dipterocarpaceae*, one new species, saprobic fungi, *Sporocadaceae*, Thailand, *Xylariomycetidae incertae sedis*

## Abstract

Leaf litter is an essential functional aspect of forest ecosystems, acting as a source of organic matter, a protective layer in forest soils, and a nurturing habitat for micro- and macro-organisms. Through their successional occurrence, litter-inhabiting microfungi play a key role in litter decomposition and nutrient recycling. Despite their importance in terrestrial ecosystems and their abundance and diversity, information on the taxonomy, diversity, and host preference of these decomposer taxa is scarce. This study aims to clarify the taxonomy and phylogeny of four saprobic fungal taxa inhabiting *Dipterocarpus alatus* leaf litter. Leaf litter samples were collected from Doi Inthanon National Park in Chiang Mai, northern Thailand. Fungal isolates were characterized based on morphology and molecular phylogeny of the nuclear ribosomal DNA (ITS, LSU) and protein-coding genes (*tub2*, *tef1-α*, *rpb2*). One novel saprobic species, *Ciliochorella dipterocarpi*, and two new host records, *Pestalotiopsis dracontomelon* and *Robillarda australiana*, are introduced. The newly described taxa are compared with similar species, and comprehensive descriptions, micrographs, and phylogenetic trees are provided.

## 1. Introduction

Leaf litter is abundantly found in forest ecosystems with a rich source of organic matter [1,2], where fungi act as key players in decomposing leaf litter by the production of extracellular enzymes [3,4,5,6]. Fungi are cosmopolitan organisms that thrive in diverse ecosystems and environments [7,8,9,10,11]. They are involved in fundamental ecological processes as pathogens, mutualists, and decomposers [12,13]. Saprobic fungi are involved in carbon cycling and nutrient recycling [7,8,9,10,11]; mediating the functioning and balance in forest ecosystems [14]. Despite their role and abundance, fungal diversity and its associations with host species are poorly studied; thus, exploring tropical ecosystems for unidentified fungal species has gained attention [15,16,17].

*Sordariomycetes* is the second-largest class in *Ascomycota* [18,19,20], with members with a cosmopolitan distribution [21,22]. They act as pathogens, endophytes [23,24], and saprobes [25]. Additionally, some taxa have been reported as opportunistic pathogens [26] and fungicolous [27]. In this group, appendage-bearing coelomycetes containing fusoid multi-septate conidia are circumscribed as pestalotioid fungi [23,28,29] and classified as *Sporocadaceae* [30]. They are characterized by their asexual morphology: acervular conidiomata, conidiogenesis, and conidia [29]. However, due to the absence of strong morphological markers for some genera, multilocus phylogenetic analyses are essential for their taxonomic placements [29,31,32,33].

Nevertheless, within appendage-bearing fungi, *Pestalotiopsis* is comprised mostly of asexual morphs, rich with cryptic species [34,35], with the morphological species delimitation taxonomically insignificant [36]. To solve this issue, in the most recent review of resolving the genus taxonomy, Maharachchikumbura et al. [23] used multilocus phylogeny based on internal transcribed spacer (ITS) region, partial translation elongation factor (*tef*1-α), and β-tubulin (*tub*2) genes coupled with morphology and set the species boundaries within the genus. On the other hand, *Ciliochorella* (*Xylariomycetidae incertae sedis*) and *Robillarda* (*Sporocadaceae*) have distinctive morphological characteristics while lacking complete molecular information for most of the species.

Assessing the tropical forest mycobiome to recover novel fungal taxa and document their host associations, life modes, abundance through seasonal variations, and ecological preference is critical in elucidating fungal biodiversity. In contrast, taxonomy to assess biodiversity is also important [37]. The present study aimed to investigate the taxonomy and phylogeny of three saprobic appendage-bearing *Sordariomycetes* from the leaf litter of *Dipterocarpus alatus* collected from Doi Inthanon National Park, Thailand. One novel species, *Ciliochorella dipterocarpi,* and two new host records, *Pestalotiopsis dracontomelon* and *Robillarda australiana*, were identified and described using morpho-molecular analysis.

## 2. Materials and Methods

### 2.1. Sample Collection and Fungal Isolation

*Dipterocarpus alatus* leaf litter was collected from Doi Inthanon National Park, Chiang Mai, Thailand. The samples were stored in separate paper bags, brought back to the laboratory, and pressed in between newspaper sheets. After two days, leaf samples were placed in a sterile moisture chamber to favor the emergence of saprobic fungal taxa. The specimens were examined using a dissecting microscope (Motic SMZ-168, Wetzlar, Germany) and a light microscope (Nikon ECLIPSE 80i, Tokyo, Japan). Single-spore isolation was carried out in potato dextrose agar (PDA) plates as described by Senanayake et al. [38]. The obtained cultures were incubated at 25 °C for 2–3 weeks with frequent observation to detect and record the sporulation and colony characteristics.

### 2.2. Morphological Observation

Freehand sectioning through the conidiomata was performed to observe the morphological characteristics (e.g., conidiomata shape, conidiomatal wall cell structure, conidiophores, conidiogenous cells, and conidia). Colony characteristics were examined and recorded after 7 days of incubation at 25 °C. Fungal fruiting bodies were mounted on water and examined with a light microscope (Nikon Eclipse E600), and digital images were captured with a Nikon DS-U2 and Cannon 750D camera (Tokyo, Japan). All measurements were made using the Tarosoft (R) Image Framework software v.0.9.0.7, and Adobe Photoshop CS6 v. 12.0 (Adobe Systems, San Jose, CA, USA) was used for making photo plates.

### 2.3. Material Deposition

All the herbarium materials were deposited in the Mae Fah Luang University Herbarium (MFLU), Chiang Rai, Thailand, and living cultures were deposited in the Mae Fah Luang University Culture Collection (MFLUCC). Faces of Fungi and Index Fungorum numbers were obtained as in Jayasiri et al. [39] and Index Fungorum [40]. The obtained sequences were deposited in the GenBank database. The illustrations and descriptions were submitted to the GMS MICROFUNGI database [41]. The novel species was introduced following the guidelines by Maharachchikumbura et al. [42].

### 2.4. DNA Extraction, PCR Amplification, and Sequencing

Genomic DNA was extracted from the margins of 2-week-old cultures grown on PDA plates at 25 °C using a Forensic DNA Kit (OMEGA, Biotek, Norcross, GA, USA), following the manufacturer’s instructions. Polymerase chain reactions (PCR) were conducted in an Applied Biosystems C1000 Touch TM Thermal Cycler with the PCR conditions specified in Table 1. The PCR reaction mixture was composed of 25 μL of Taq DNA polymerase, 2 μL of genomic DNA, 2 μL (20 μM) of each primer, and 19 μL of double-distilled H_2_O. PCR products were visualized via agarose gel electrophoresis with 1% agarose gel under the Biorad gel documentation system (Hercules, CA, USA). Sequencing of PCR products was carried out bi-directionally by Biogenomed, Seoul, Korea. All the sequences generated in this study were deposited in GenBank.

### 2.5. Phylogenetic Analyses

The obtained sequence chromatograms were checked with Chromas 2.6.6 (Technelysium Pty Ltd., South Brisbane, Australia), and the low-quality regions were trimmed. The sequences were subjected to BLASTn searches against the NCBI nucleotide non-redundant databases, with the option “sequences from type material” selected. Separate data sets were used to perform phylogenetic analyses for *Pestalotiopsis*, *Ciliochorella*, and *Robillarda*. Reference sequences were obtained from recent literature [50,51] and downloaded from GenBank (www.ncbi.nlm.nih.gov/genbank/ (accessed on 18 November 2022)) (Table 2, Table 3 and Table 4). Taxon sampling for *Pestalotiopsis* was performed by selecting the type strains and a duplicate strain if available from all the recorded species, excluding unverified sequences, to obtain better topology for the phylogenetic tree. The single locus of each data set was aligned by MUSCLE [52] implemented in MEGA (v. 7.0.26), applying the default settings. The aligned sequences were automatically trimmed using trimAl 1.2 [53] under the default -*gappyout* option for all loci and concatenated using BioEdit v. 7.0.5.2 [54]. Phylogenetic analyses were based on maximum likelihood (ML), maximum parsimony (MP), and Bayesian inference (BI) posterior probability (PP). ML, MP, and BI were performed for *Pestalotiopsis*, while *Ciliochorella*, and *Robillarda* were resolved using model-based methods (ML, BI) following the previous literature [23,29,50]. ML and BI were performed in the CIPRES Science Gateway platform [55]. ML was executed by RAxML-HPC2 on XSEDE v. 8.2.8 [56,57], with 1000 bootstrap replicates under the GTRGAMMA nucleotide substitution model.

Bayesian analysis was performed using MrBayes v. 3.2.7a on XSEDE [58] Markov Chain Monte Carlo (MCMC) sampling to calculate the posterior probabilities [59,60], under the nucleotide evolutionary models calculated by jModelTest v.2.1.6 (Table 5). Then, 2 parallel independent runs with 6 MCMCs were run for 15,000,000 (*Pestalotiopsis*) and 3,000,000 generations (*Ciliochorella* and *Robillarda*), with trees sampled every 1000th generation. Twenty-five percent of the trees representing the burn-in phase were discarded, and the remaining trees were used to calculate the PP in the majority-rule consensus tree.

Maximum-parsimony trees were generated by PAUP v4.0b10 [59], using the heuristic search option with 1000 random sequence additions, with Maxtrees set to 1000. Branches of zero length were collapsed, and all maximum parsimony trees were saved. Descriptive tree statistics for parsimony-tree length (TL), consistency index (CI), retention index (RI), relative consistency index (RC), and homoplasy index (HI) were calculated following the Kishino–Hasegawa test (KHT) criteria [61].

The phylogenetic trees were visualized and exported using FigTree v.1.4.0 [62], and the phylograms were edited and annotated in Microsoft PowerPoint (2013) and Adobe Photoshop CS6.

## 3. Results

### 3.1. Phylogenetic Analyses

**Figure 1:**
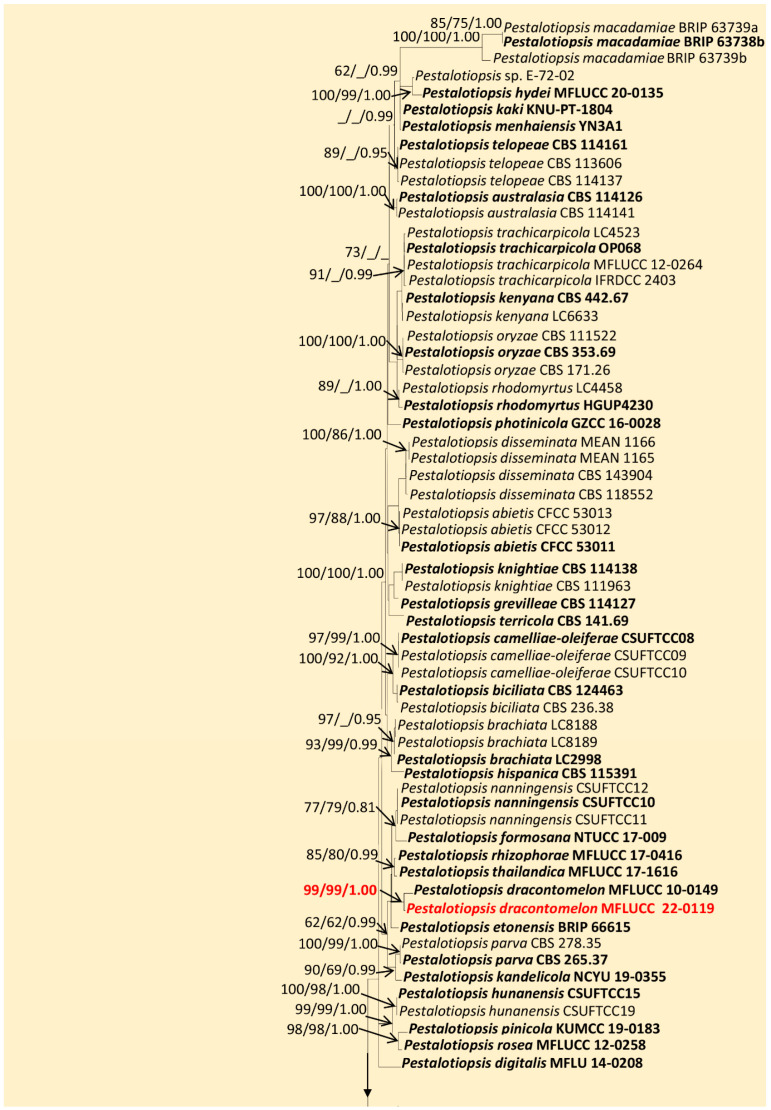

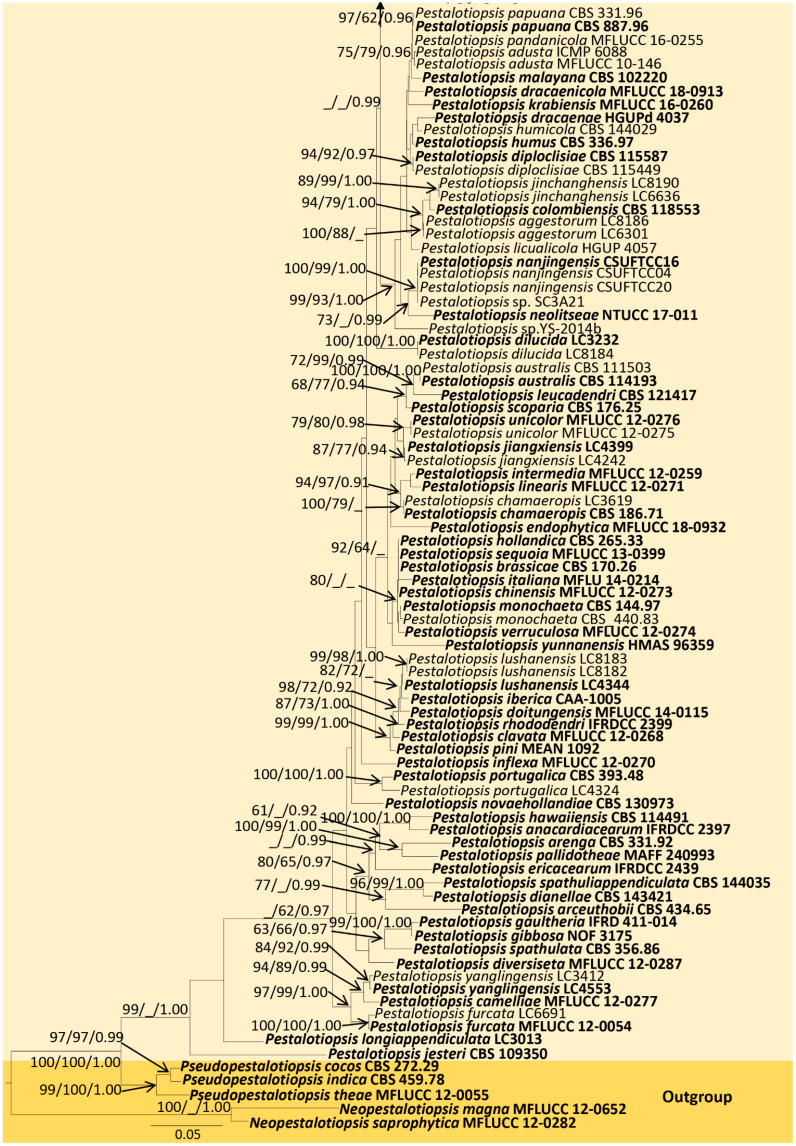
Maximum-likelihood consensus tree inferred by RAxML analysis of combined ITS, *tub2,* and *tef1-a* sequence data for *Pestalotiopsis*. Bootstrap support values for ML ≥ 60%, MP ≥ 60%, and PP ≥ 0.90 are given at the nodes (ML/MP/PP). The tree is rooted in *Neopestalotiopsis magna* (MFLUCC 12-0652) and *N. saprophytica* (MFLUCC 12-0282). Type and ex-type strains are shown in bold, and newly identified strain is in red.

The combined alignment (ITS, *tef1-α*, and *tub2*) of *Pestalotiopsis* comprised 146 taxa, including 5 outgroup taxa. The best-scoring ML tree (Figure 1) had an ML optimization likelihood value of −16,987.384789. There were 1,025 distinct alignment patterns with 26.01% undetermined characters or gaps. The estimated base frequencies were as follows: A = 0.238425, C = 0.294335, G = 0.217098, and T = 0.250142; substitution rates, AC = 1.142015, AG = 3.088078, AT = 1.117450, CG = 0.907663, CT = 4.297777, and GT = 1.000000; gamma distribution shape parameter *α* = 0.286165; and tree length = 1.911747. The MP analysis for the combined dataset had 576 parsimony-informative, 1389 constant, and 259 parsimony-uninformative characters and yielded the single most parsimonious tree with the parameters TL = 2471, CI = 0.492, RI = 0.791, RC = 0.389, and HI = 0.508. The Bayesian analysis calculated the average standard deviation of split frequencies at the end of 15,000,000 MCMC generations with a stop value of 0.009. The ML, MP, and BI trees were similar in topology. The resulting phylogenetic tree from the concatenated alignment resolved our *Pestalotiopsis* isolate into a novel host record, *P. dracontomelon* (MFLUCC 22-0119), with strong statistical support (99% ML, 99% MP, 1.00 PP) (Figure 1).

**Figure 2:**
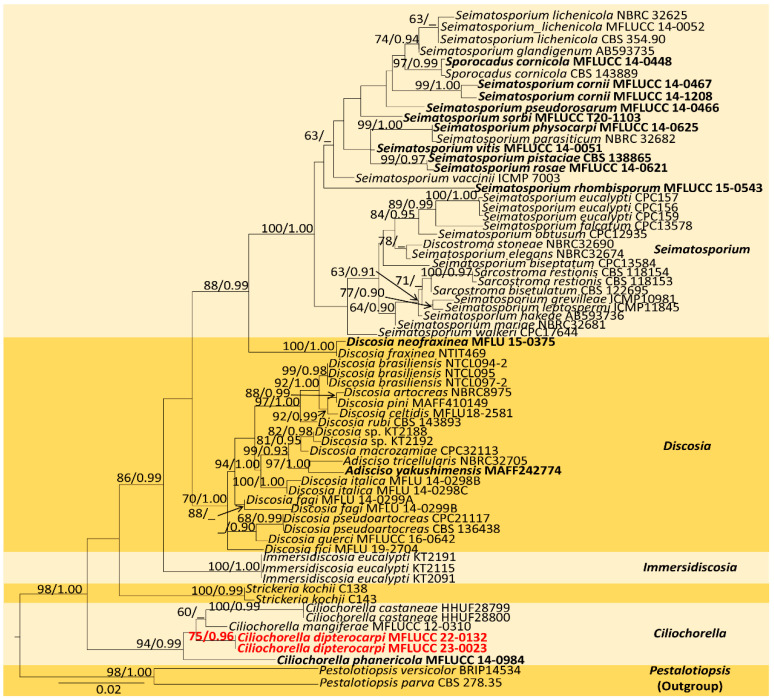
Maximum-likelihood consensus tree inferred by RAxML analysis of combined LSU, ITS, and *tub2* sequence data. Bootstrap support values for ML ≥ 60% and PP ≥ 0.90 are given at the nodes (BS/PP). The tree is rooted with *Pestalotiopsis versicolor* (BRIP14534) and *P. parva* (CBS 278.35). The type species are shown in bold, and the newly identified strain is in red.

The combined sequence alignment (LSU, ITS, and *tub2*) of *Ciliochorella* comprised 71 taxa, including *Pestalotiopsis versicolor* (BRIP 14534) and *P. parva* (CBS 278.35) as the outgroup taxa. The matrix had 599 distinct alignment patterns with 34.06% undetermined characters or gaps. The ML analysis of the combined dataset yielded a best-scoring tree with a final ML optimization likelihood value of −8593.236182. The estimated base frequencies were as follows: A = 0.252388, C = 0.224809, G = 0.259858, and T = 0.262945; substitution rates, AC = 1.255639, AG = 2.934468, AT = 1.259383, CG = 1.113458, CT = 5.503206, and GT = 1.000000; gamma distribution shape parameter *α* = 0.120262; and tree length = 0.748074. In the Bayesian analysis, the average standard deviation of split frequencies at the end of 3,000,000 MCMC generations was calculated with a stop value of 0.009935. The ML and BI trees were similar in topology. The resulting phylogenetic tree from the concatenated alignment resolved *C. dipterocarpi* (MFLUCC 22-0132) isolate into a well-supported distinct lineage with strong statistical support (94% ML, 0.95 PP) (Figure 2).

**Figure 3:**
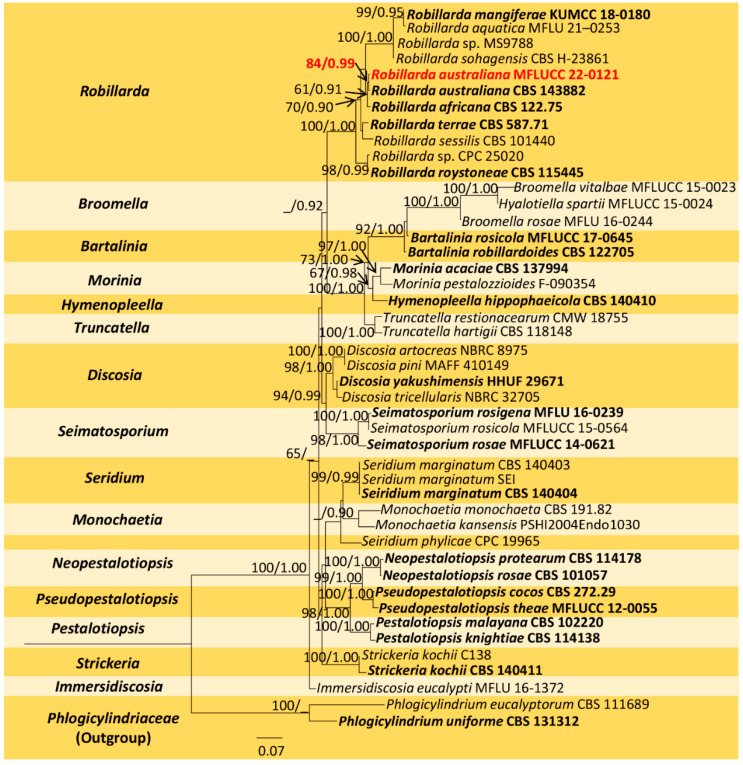
Maximum-likelihood consensus tree inferred by RAxML analysis of combined ITS, LSU, *rpb2*, and *tef1-a* sequence data. Bootstrap support values for ML ≥ 60% and PP ≥ 0.90 are given at the nodes (BS/PP). The tree is rooted with *Phlogicylindrium eucalyptorum* (CBS 111689) and *P. uniforme* (CBS 131312). Type species are shown in bold, and the newly generated strain is in red.

The combined sequence alignment (ITS, LSU, *rpb2*, and *tef1-α*) of *Robillarda* comprised 45 taxa, including *Phlogicylindrium eucalyptorum* (CBS 111689) and *P. uniforme* (CBS 131312) as the outgroup taxa. The matrix had 1482 distinct alignment patterns with 44.64% undetermined characters or gaps. The ML analysis of the combined dataset yielded a best-scoring tree with a final ML optimization likelihood value of −26214.966097. The estimated base frequencies were as follows: A = 0.250239, C = 0.240486, G = 0.252222, and T = 0.257053; substitution rates, AC = 1.409959, AG = 3.149767, AT = 1.321223, CG = 1.040419, CT = 5.807309, and GT = 1.000000; gamma distribution shape parameter *α* = 0.244419; and tree length = 3.825788. In the Bayesian analysis, the average standard deviation of split frequencies at the end of 3,000,000 MCMC generations was calculated with a stop value of 0.009951. The ML and BI trees were similar in topology. The resulting phylogenetic tree from the concatenated alignment grouped the isolate MFLUCC 22-0121 with the ex-type strain *R. australiana* (CBS 143882) and formed a well-supported clade with strong statistical support (84% ML, 0.99 PP) (Figure 3).

### 3.2. Taxonomy

#### 3.2.1. *Pestalotiopsis dracontomelon* Maharachch. and K.D. Hyde

**Figure 4:**
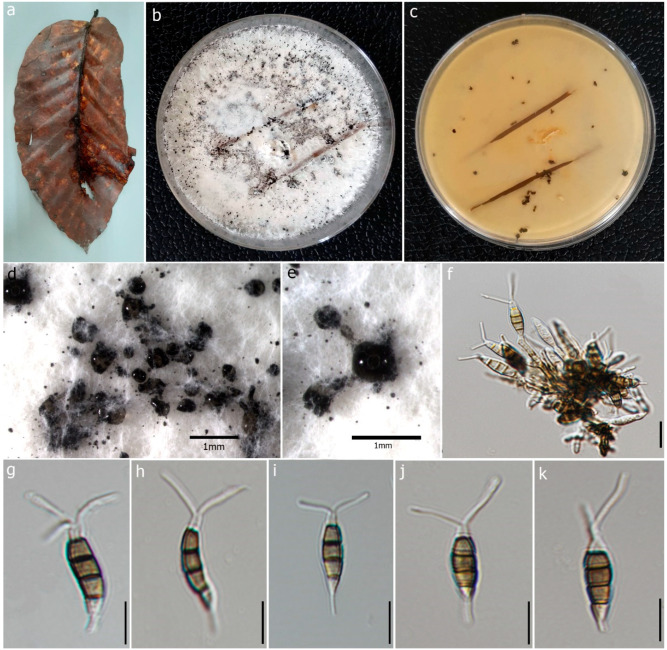
*Pestalotiopsis dracontomelon* (MFLU 22-0195, new host record). (**a**) Dead leaf of *Dipterocarpus alatus*. (**b**,**c**) Upper and reverse view of the colonies on PDA. (**d**,**e**) Conidiomata on PDA. (**f**) Conidiogenous cells with developing conidia. (**g**–**k**) Conidia. Scale bars: (**d**,**e**) = 1 mm, (**f**) = 5 μm, (**g**–**k**) = 10 μm.

Index Fungorum number: IF550943; Faces of Fungi number: FoF00457 (Figure 4)

**Description:** Saprobic on dead leaves of *D. alatus.* **Sexual morph**: Undetermined. **Asexual morph:** Conidiomata 60–75 × 40–60 μm (x = 68 × 50 μm, n = 20), pycnidial, black, globose, scattered on PDA culture media. Conidiophores subcylindrical, branched base with 2–3 septa. Conidiogenous cells 4–10 × 1.7–2.3 μm (x = 6.05 × 1.98 μm, n = 10) with percurrent proliferation of 1–2 times, integrated, cylindrical. Conidia 14–22 × 4–6 μm (x = 18 × 5 μm, n = 50), straight to slightly curved, fusoid, 4-septate; basal cell 2–5 μm long, conic, truncate base, hyaline, thin-walled; three median cells 10–13 μm (x = 12 μm) long, doliform or subcylindrical, olivaceous, concolorous and verrucose wall; second cell from the base 3–5 μm (x = 3 μm); third cell 2–4 μm (x = 3 μm); fourth cell 3–5 μm (x = 4 μm); apical cell 2–3 μm long, subcylindrical, hyaline, thin-walled, rugose; 2 appendages 10–19 μm long (x = 14 μm), tubular, filiform, unbranched, flexuous, appendages arise from apical crest; single basal appendage 2–6 μm long (x = 4 μm), unbranched, tubular, centric.

**Culture characteristics:** Colonies on PDA reaching 4.5 cm diam. after 7 days at 25 °C, aerial, whitish, smooth edge, entire margin, with gregarious black spore masses. Colonies creamy white from above and reverse without producing pigmentation on media.

**Material examined:** Thailand, Chiang Mai Province, Doi Inthanon National Park, (18.5356° N, 98.5221° E), on dead leaves of *D. alatus* (*Dipterocarpaceae*), 25 October 2021, Milan C. Samarakoon, D6a (MFLU 22-0195); living culture, MFLUCC 22-0119.

**Known host:** Pathogenic on leaves of *Dracontomelon mangifera* (*Anacardiaceae*) [63], leaf spots of *Podocarpus* sp. (*Podocarpaceae*) [27], saprobic on *D. alatus* leaf litter (this study).

**Known distribution:** Thailand.

**Notes:***Pestalotiopsis dracontomelon* was introduced by Liu et al. [63], as a pathogenic species of *Dracontomelon mangiferum* in Thailand. Our isolate (MFLUCC 22-0119) shares the morphological characteristics of the type of *P. dracontomelon* (MFLUCC 10-0149), having septate conidiophores, conidiogenous cells with percurrent proliferation, 4-septate conidia, 2–3 tubular apical appendages, and a single centric basal appendage [63] with slight variations in the sizes of conidiogenous cells and conidia (x = 18 × 5 μm vs. 20 × 6.5 μm). According to the multilocus phylogeny (ITS, *tub2,* and *tef1-α*), our isolate clustered with the type *P. dracontomelon* with solid statistical support (99% ML, 99% MP, 1.00 PP). Thus, we introduce it as a new host record from *D. alatus* in Thailand.

#### 3.2.2. *Ciliochorella dipterocarpi* Samaradiwakara, Lumyong and K.D. Hyde, sp. nov.

**Figure 5:**
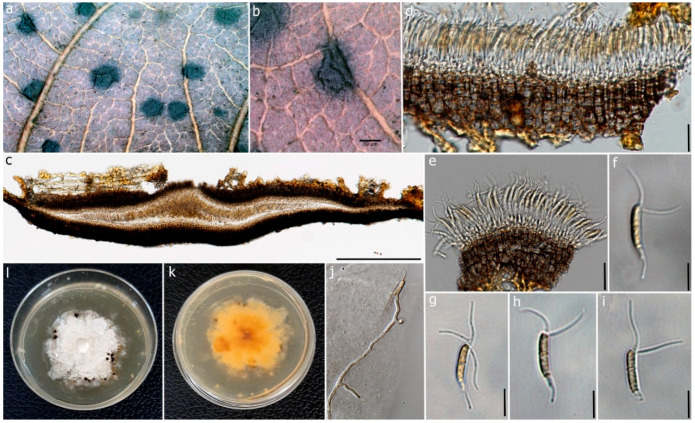
*Ciliochorella dipterocarpi* (MFLUCC 22-0132, holotype). (**a**) Conidiomata on the leaf. (**b**) Close-up of conidioma. (**c**) Vertical section of the conidioma. (**d**) Peridium. (**e**) Conidiogenous cells with developing conidia. (**f**–**i**) Conidia. (**j**) Germinating conidium. (**k**,**l**) Reverse and front view of the colonies on PDA. Scale bars: (**b**) = 50 μm, (**c**) = 50 μm, (**d**) = 15 μm, (**e**) = 10 μm, (**f**–**i**) = 15 μm, (**j**) = 20 μm.

Index Fungorum number: IF900181; Faces of Fungi number: FoF13617 (Figure 5).

**Etymology.** The epithet *“dipterocarpi”* refers to the host genus.

**Holotype.** MFLU 22-0197.

**Description:** Saprobic on dead leaves of *D. alatus*. **Sexual morph**: Undetermined. **Asexual morph**: Coelomycetous. Conidiomata 120–150 μm high, 650–800 μm diam., acervulus, superficial or semi-immersed, mostly solitary, unilocular, horizontally fusiform. Peridium 20–35 μm wide, 6–8 layered, cells of *textura angularis*, composed of thin-walled pale-brown inner layer, thick-walled, dark brown to black outer layer. Conidiophores reduced to conidiogenous cells. Conidiogenous cells 0.6–1.3 μm (x = 0.95 μm, n = 30), hyaline to pale brown, smooth. Conidia 9–18 × 1–3 μm (x = 14 × 2 µm, n = 50), fusiform to naviculate, allantoid to sub-cylindrical, slightly curved, slightly guttulate, 1-septate, septum thicker and slightly darker, hyaline to pale brown, thick-walled, narrow towards the base, bearing appendages on both apical and basal ends; wide middle cell, basal end narrowly obconic, with truncate base terminated into a filiform appendage; apical end forked, hyaline at apex and sub-hyaline below with 2–3 apical appendages. Appendages tubular, filiform, flexuous; appendage on apical cell 12–15 μm (x = 13 μm) long, 2–3 tubular, unequal; basal appendage 8–17 µm (x = 10 µm) long, single.

**Culture characteristics:** Colonies on PDA, reaching 3.5 cm diam. after 7 days at 25 °C. *Mycelium* dense, flat, circular, or round, with smooth, erose, entire margin. Colonies white to cream from front, pale yellow from reverse.

**Material examined:** Thailand, Chiang Mai Province, Doi Inthanon National Park (18.5356° N, 98.5221° E), on dead leaves of *D. alatus* (*Dipterocarpaceae*), 25 October 2021, Milan C. Samarakoon, NP009 (MFLU 22-0197, Holotype), ex-type living culture, MFLUCC 22-0132.

**Notes:** *Ciliochorella* belongs to *Xylariomycetidae incertae sedis* in *Sordariomycetes*, and it produces appendage-bearing conidia [29]. The multilocus phylogeny indicates that our isolate (MFLUCC 22-0132) has a precise placement and clusters basal to *C. mangiferae* (MFLUCC 12-310), *C. castaneae* (HHUF28800), and *C. castaneae* (HHUF28799), with 81% ML and 0.95 PP statistical support (Figure 2). In the BLAST search in GenBank, the closest match for the ITS region of *C. dipterocarpi* (MFLUCC 22-0132) was *C. phanericola* (MFLUCC 12-310) with 99.45% similarity across 95% query coverage, LSU with a 99.77% similarity to *C. phanericola* (MFLUCC 12-310) across 97% query coverage, and *tub2* with a 98.97% similarity to *Ciliochorella* sp. (MFLUCC 12-310) across 90% query coverage. The major difference in base pairs of the ITS region between *C. dipterocarpi* (MFLUCC 22-0132) and *C. castaneae* was 1.18% (10/841), and between *C. dipterocarpi* and *C. mangiferae*, it was 1.71% (7/408). The base pair difference of the *tub2* between *C. dipterocarpi* and *C. phanericola* was 11.93% (37/310). The values agree with the new species concept outlined by Maharachchikumbura et al. [42].

Morphologically, *C. dipterocarpi* can be distinguished from other *Ciliochorella* species based on having conidiogenous cells bearing conidia with mostly 2-septate, slightly guttulate, and shorter apical appendages, 12–15 μm (x = 13 μm) long. In comparison, *C. castaneae* has conidia that are 0–2-septate with branched conidiophores [64], whereas *C. mangiferae* and *C. phanericola* have conidia in the range of 9–18 ×1–3 μm [50,65]. *Ciliochorella dipterocarpi* conidial sizes include 9–18 × 1–3 μm, with 2-3 apical appendages, and 12–15 μm long, with single basal appendage 8–17 µm long. In this study, we isolated *C. dipterocarpi* from the leaf litter of *D. alatus*, and considering the variations in morphological characteristics and phylogenetic placement, *C. dipterocarpi* (MFLUCC 22-0132) is introduced as a novel species.

#### 3.2.3. *Robillarda australiana* F. Liu, L. Cai & Crous

**Figure 6:**
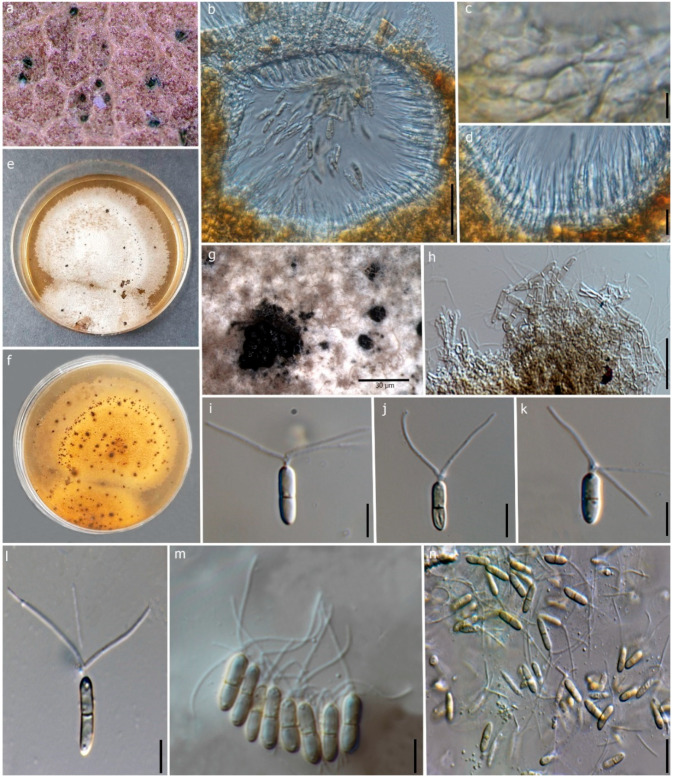
*Robillarda australiana* (MFLU 22-0198, new host record). (**a**) Conidiomata on the leaf. (**b**) Vertical section of the conidioma. (**c**) Conidiomata wall at the base. (**d**) Conidiogenous cells and developing conidia. (**e**,**f**) Front and reverse view of the colonies on PDA. (**g**) Conidiomata on PDA. (**h**) Conidiophores and conidiogenous cells. (**i**–**n**) Conidia. Scale bars: (**b**) = 50 μm, (**c**) = 15 μm, (**d**) = 5 μm, (**g**) = 30 μm, (**h**–**n**) = 10 μm.

Index Fungorum number: IF828387; Faces of Fungi Number: FoF13618 (Figure 6)

**Description:** Saprobic on dead leaves of *D. alatus*. **Sexual morph**: Undetermined. **Asexual morph**: Coelomycetous. Conidiomata 120–150 × 100–150 μm (x = 137 × 119 μm, n = 10), mostly erumpent, semi-immersed, pycnidial, mostly solitary, irregular shape, thick-walled, black. Conidiomatal wall 10–15 μm wide, thick-walled at upper wall, 1-2 layers of dark brown outer cells, inner cells hyaline, arranged in *textura angularis*. Conidiophores reduced to conidiogenous cells. Conidiogenous cells 4–10 × 2–4 μm (x = 7 × 3 μm, n = 10), guttulate, smooth, thin-walled, hyaline, discrete, irregular or ampulliform, lageniform, 2–3 distinct, small protuberances at the apex. Conidia 7–10 × 2–3 μm (x = 8 × 2.5 μm, n = 25), guttulate, cylindrical, 1-septate, slightly constricted at the median septum, smooth, straight, hyaline to pale brown, symmetrical, cylindrical apical cell modified into three appendages. Apical appendages unbranched, 3 divergent branches, attenuated at the apex, 9–17 μm (x = 14 μm, n = 15) long; basal appendages absent.

**Culture characteristics:** Conidia germinated on PDA within 24 h. Colonies on PDA, reaching 3 cm after 14 days at 25 °C. *Mycelia* superficial, flat, entire margin, surface rough, form black pycnidia after one month of incubation. Colonies white to light brown from front, pale yellow from reverse without producing pigmentation.

**Material examined:** Thailand, Chiang Mai Province, Doi Inthanon National Park, (18.5356° N, 98.5221° E), on dead leaves of *D. alatus*, 25 October 2021, Milan C. Samarakoon, NPSC 04 (MFLU 22-0198) living culture, MFLUCC 22-0121.

**Known hosts:** *D. alatus* (this study).

**Known distribution:** Australia, Thailand.

**Notes:** Phylogeny shows that our isolate (MFLUCC 22-0121) clusters with the type *R. australiana* (CBS 143882) with 84% ML and 0.99 BYPP (Figure 3). Both isolates share similar morphology, having conidia without constriction at the median septum with larger apical cells (*R. australiana* MFLUCC 22-0121, 8 × 2.5 μm vs. CBS 143882 11 × 2 μm) [29]. Therefore, we introduce our collection as a new host record of *R. australiana* from dead leaves of *D. alatus* in Thailand.

## 4. Discussion

Appendages are informative morphological characteristics in delineating fungal species [28]. Additionally, they support the adherence of spores to their substrate and dispersal, characterizing important ecological functions [28]. In this study, we used a polyphasic approach for the species boundaries of novel collections of leaf-litter-inhabiting fungi [42,66,67,68]. With this approach, out of three appendage-bearing *Sordariomycetes* collected from Doi Inthanon National Park, northern Thailand, one was placed as *Ciliochorella dipterocarpi* sp. nov, and two (*P. dracontomelon* and *R. australiana*) were placed as new records on *D. alatus*.

*Ciliochorella* is characterized by its distinct morphological characteristics [64,65] and molecular phylogeny based on the ITS, LSU, and *tub2* sequence data [50]. Nevertheless, many species and isolates lack the sequence data for one or more loci, which may cause biases in the tree topology and phylogenetic placement [69,70,71,72] [e.g., 71,72,73,74]. Still, our newly introduced taxon *C. dipterocarpi* is clustered sister to *C. mangiferae* (MFLUCC 12-0310) (Figure 2), which has all the loci with available sequence data (Table 3). Like *C. dipterocarpi*, *Ciliochorella* species have been mainly reported as saprobes on various plant litter. In the present study, using a taxonomic approach (Figure 2), we expand the diversity of *Ciliochorella* with the introduction of *C. dipterocarpi*, isolated from the dead leaves of *D. alatus*.

Regarding the new litter host association of *P. dracontomelon* (MFLUCC 22-0119) and *D. alatus*, *Pestalotiopsis* species have been reported as endophytes producing important bioactive secondary metabolites, pathogens on several economically important crops, and saprobes on different plant litter [36,73,74,75,76]. Since the last detailed morphology and molecular phylogeny update [23,24], several species have been introduced, and around 100 *Pestalotiopsis* species are currently accepted [29,30,77,78,79]. However, the taxonomic placement is doubtful, mainly due to the lack of strong morphological characteristics [36] and the poor resolution of the employed molecular markers (ITS, *tub2*, and *tef1-α*), which presumably identify species rather than populations within the genus [80]. In our analysis, as taxon sampling is fundamental for the taxonomic placement and delimitation [81,82], to obtain better phylogenetic resolution (Figure 1), we excluded distantly related species and those with inconsistent sequence data and included only representative type strains (Table 2). Thus, a taxonomic revision of this genus is urgently needed, especially using the polyphasic approach with additional genomic regions [24,42,80,83,84].

Unlike *Pestalotiopsis, Robillarda* is characterized by its distinct morphological features [85,86], coupled whenever possible with the multilocus phylogeny of the ITS, *rpb2*, *tub2*, and *tef1-α* barcodes [29]. Nevertheless, out of its 41 species listed in the Index Fungorum database [40], only 8 have molecular data available [29,67,85,87,88]. *Robillarda* species are found as saprobic on decaying leaves in terrestrial and aquatic habitats, dust particles, and soil [85]. Here, we contribute with the expansion of the host and geographical record of *R. australiana*, which was found as a saprobe on decaying leaves of *D. alatus* (Figure 6) at Doi Inthanon National Park, Thailand. This species was only reported in Australia on unidentified plant litter [29].

Many fungal species have been discovered in Thailand, using polyphasic approaches [89]. As evident from the present study, much work remains to be done in this regard. This includes examining unstudied areas, such as protected national parks and hosts, to address whether fungi are host-specific and at which level [13]. Tennakoon et al. [90] contributed significantly to this matter. This is important in predicting the number of species [19] and in assessing the diversity in a given area or ecosystem [91], which have the same significance [92]. Currently, only approximately 10% of the 2.2–3.8 million species estimated by Hawksworth and Lucking [93] have been described [13,16,94], highlighting the potential for unraveling novel fungal taxa in largely untapped ecosystems. In this regard, this study contributes to the above topics, and the number of teleomorph species of Ascomycota estimated by Senanayake et al. [95] was between 1.37 and 2.56 million. Finally, the findings of the present study provide additional insights into fungal diversity in pristine tropical environments, as predicted by Hyde et al. [89]. However, given the different lifestyles of the species, elucidating their evolution, host and lifestyle shifting, and environmental adaptations is needed for a better understanding of their roles [13]. 

## 5. Conclusions

Ecological preference and evolutionary relationships can support the emergence of novel species in contrasting life modes [4,13]. This hypothesis expands the scope of saprobic fungal habitats and their host preferences [4,13]. This scenario is supported by identifying saprobic lifestyles of different genera from the present study. Moreover, we also identified fungal species associated with novel hosts and geographical locations (*P. dracontomelon* and *R. australiana*), along with the novel species *C. dipterocarpi.*

## Figures and Tables

**Table 1:** Primers and PCR conditions used in this study.

Gene/Loci	Primers	PCR Conditions	Reference
ITS	ITS5/ITS4	Initial denaturation 94 °C for 3 min, following 35 × cycles of denaturation 94 °C for 30 s, annealing 55 °C for 50 s, elongation 72 °C for 1.30 min, and a final extension 72 °C for 10 min.	[43]
LSU	LR0R/LR5	Initial denaturation 94 °C for 3 min, following 35 × cycles of denaturation 94 °C for 30 s, annealing 55 °C for 50 s, elongation 72 °C for 1.30 min, and a final extension 72 °C for 10 min.	[44,45]
*tef-1a*	EF728F/EF2 and EF728F/EF-986R	Initial denaturation 94 °C for 3 min, followed by 35 × cycles of denaturation 94 °C for 30 s, annealing 58.5 °C for 1.30 min, elongation 72 °C for 1.20 min, and a final extension 72 °C for 5 min.	[46]
*tub2*	BT-2a/BT-2b and T1/T22	Initial denaturation 94 °C for 3 min, followed by 35 × cycles of denaturation 9 °C for 30 s, annealing 58.5 °C for 1.30 min, elongation 72 °C for 1.20 min, and a final extension 72 °C for 5 min.	[47]
*rpb2*	fRPB25F/fRPB2-7cR	Initial denaturation 95 °C for 5 min, followed by 40 × cycles of denaturation 95 °C for 15 s, annealing 56 °C for 50 s, elongation 72 °C for 2 min, and a final extension 72 °C 10 min.	[48,49]

**Table 2:** Newly generated sequences in this study are indicated in bold. * indicates type strains.

Species	Isolate	Substrate/Host	Country	ITS	*tub2*	*tef1-a*
*Pestalotiopsis abietis*	CFCC 53011 *	*Abies fargesii*	China	MK397013	MK622280	MK622277
*P. abietis*	CFCC 53012	*Abies fargesii*	China	MK397014	MK622281	MK622278
*P. abietis*	CFCC 53013	*Abies fargesii*	China	MK397015	MK622282	MK622279
*P. adusta*	ICMP 6088 *	Refrigerator door	Fiji	JX399006	JX399037	JX399070
*P. adusta*	MFLUCC 10-0146	*Syzygium* sp.	Thailand	JX399007	JX399038	JX399071
*P. aggestorum*	LC6301 *	*Camellia sinensis*	China	KX895015	KX895348	KX895234
*P. aggestorum*	LC8186	*Camellia sinensis*	China	KY464140	KY464160	KY464150
*P. anacar- diacearum*	IFRDCC 2397 *	*Mangifera indica*	China	KC247154	KC247155	KC247156
*P. arceuthobii*	CBS 434.65 *	*Arceuthobium campylopodum*	USA	KM199341	KM199427	KM199516
*P. arenga*	CBS 331.92 *	*Arenga undulatifolia*	Singapore	KM199340	KM199426	KM199515
*P. australasia*	CBS 114126 *	*Knightia* sp.	New Zealand	KM199297	KM199409	KM199499
*P. australasia*	CBS 114141	*Protea* sp.	New South Wales	KM 199298	KM199410	KM199501
*P. australis*	CBS 111503	*Protea neriifolia*	South Africa	KM199331	KM199382	KM199557
*P. australis*	CBS114193 *	*Grevillea* sp.	NewSouth Wales	KM199332	KM199383	KM199475
*P. biciliata*	CBS 124463 *	*Platanus* × *hispanica*	Slovakia	KM199308	KM199399	KM199505
*P. biciliata*	CBS 236.38	*Paeonia* sp.	Italy	KM199309	KM199401	KM199506
*P. brachiata*	LC2998 *	*Camellia* sp.	China	KX894933	KX895265	KX895150
*P. brachiata*	LC8188	*Camellia* sp.	China	KY464142	KY464162	KY464152
*P. brachiata*	LC8189	*Camellia* sp.	China	KY464143	KY464163	KY464153
*P. brassicae*	CBS 170.26 *	*Brassica napus*	New Zealand	KM199379	NA	KM199558
*P. camelliae*	MFLUCC 12-0277 *	*Camellia japonica*	China	JX399010	JX399041	JX399074
*P. camelliae-oleiferae*	CSUFTCC08 *	*Camellia oleifera*	China	OK493593	OK562368	OK507963
*P. camelliae-oleiferae*	CSUFTCC09	*Camellia oleifera*	China	OK493594	OK562369	OK507964
*P. camelliae-oleiferae*	CSUFTCC10	*Camellia oleifera*	China	OK493595	OK562370	OK507965
*P. chamaeropis*	CBS 186.71 *	*Chamaerops humilis*	Italy	KM199326	KM199391	KM199473
*P. chamaeropis*	LC3619	*Camellia* sp.	China	KX894991	KX895322	KX895208
*P. clavata*	MFLUCC 12-0268 *	*Buxus* sp.	China	JX398990	JX399025	JX399056
*P. colombiensis*	CBS 118553 *	*Eucalyptus eurograndis*	Colombia	KM199307	KM199421	KM199488
*P. digitalis*	MFLU 14-0208 *	*Digitalis purpurea*	New Zealand	KP781879	KP781883	NA
*P. dilucida*	LC3232 *	*Camellia sinensis*	China	KX894961	KX895293	KX895178
*P. dilucida*	LC8184	*Camellia sinensis*	China	KY464138	KY464158	KY464148
*P. diploclisiae*	CBS 115449	*Psychotria tutcheri*	China	KM199314	KM199416	KM199485
*P. diploclisiae*	CBS 115587 *	*Diploclisia glaucescens*	China	KM199320	KM199419	KM199486
*P. disseminata*	CBS 118552	*Eucalyptus botryoides*	New Zealand	MH553986	MH554652	MH554410
*P. disseminata*	CBS 143904	*Persea americana*	New Zealand	MH554152	MH554825	MH554587
*P. disseminata*	MEAN 1165	*Pinus pinea*	Portugal	MT374687	MT374712	MT374699
*P. disseminata*	MEAN 1166	*Pinus pinea*	Portugal	MT374688	MT374713	MT374700
*P. diversiseta*	MFLUCC 12-0287 *	*Rhododendron* sp.	China	JX399009	JX399040	JX399073
*P. doitungensis*	MFLUCC 14-0115 *	*Dendrobium* sp.	Thailand	MK993574	MK975837	MK975832
*P. dracaenicola*	MFLUCC 18-0913 *	*Dracaena* sp.	Thailand	MN962731	MN962733	MN962732
*P. dracontomelon*	MFLUCC 10-0149 *	*Dracontomelon dao*	Thailand	NR_168755	NA	NA
***P. dracontomelon***	**MFLUCC 22-0119**	***Dipterocarpus alatus***	**Thailand**	**OP905676**	**OQ127639**	**OQ127638**
*P. endophytica*	MFLUCC 18-0932 *	*Magnolia garrettii*	Thailand	MW263946	NA	MW417119
*P. ericacearum*	IFRDCC 2439 *	*Rhododendron delavayi*	China	KC537807	KC537821	KC537814
*P. etonensis*	BRIP 66615 *	*Sporobolus jacquemontii*	Australia	MK966339	MK977634	MK977635
*P. formosana*	NTUCC 17-009 *	On dead grass	China	MH809381	MH809385	MH809389
*P. furcata*	MFLUCC 12-0054 *	*Camellia sinensis*	Thailand	JQ683724	JQ683708	JQ683740
*P. furcata*	LC6691	*Camellia sinensis*	China	KX895030	KX895363	KX895248
*P. gaultheria*	IFRD 411-014 *	*Gaultheria forrestii*	China	KC537805	KC537819	KC537812
*P. gibbosa*	NOF 3175 *	*Gaultheria shallon*	Canada	LC311589	LC311590	LC311591
*P. grevilleae*	CBS 114127 *	*Grevillea* sp.	Australia	KM199300	KM199407	KM199504
*P. hawaiiensis*	CBS 114491 *	*Leucospermum* sp.	Hawaii	KM199339	KM199428	KM199514
*P. hollandica*	CBS 265.33 *	*Sciadopitys verticillata*	Netherlands	KM199328	KM199388	KM199481
*P. hispanica*	CBS 115391 *	*Protea* cv. ‘Susara’	Spain	MH553981	MH554640	MH554399
*P. humus*	CBS 336.97 *	Soil	Papua New Guinea	KM199317	KM199420	KM199484
*P. hunanensis*	CSUFTCC15 *	*Camellia oleifera*	China	OK493600	OK562375	OK507970
*P. hunanensis*	CSUFTCC19	*Camellia oleifera*	China	OK493601	OK562376	OK507971
*P.hydei*	MFLUCC 20-0135 *	*Litsea petiolata*	Thailand	MW266063	MW251112	MW251113
*P. iberica*	MUM:21.02	*Pinus sylvestris*	Spain	MW732250	MW759034	MW759037
*P. inflexa*	MFLUCC 12-0270 *	Unidentified tree	China	JX399008	JX399039	JX399072
*P. intermedia*	MFLUCC 12-0259 *	Unidentified tree	China	JX398993	JX399028	JX399059
*P. italiana*	MFLU 14-0214 *	*Cupressus glabra*	Italy	KP781878	KP781882	KP781881
*P. jesteri*	CBS 109350 *	*Fragraea bodenii*	Papua New Guinea	KM199380	KM199468	KM199554
*P. jiangxiensis*	LC4242	*Eurya* sp.	China	KX895035	KX895327	KX895213
*P. jiangxiensis*	LC4399 *	*Camellia* sp.	China	KX895009	KX895341	KX895227
*P. jinchanghensis*	LC6636 *	*Camellia sinensis*	China	KX895028	KX895361	KX895247
*P. jinchanghensis*	LC8190	*Camellia sinensis*	China	KY464144	KY464164	KY464154
*P. kandelicola*	NCYU 19-0355 *	*Kandelia candel*	China	MT560723	MT563100	MT563102
*P. kenyana*	CBS 442.67 *	*Coffea* sp.	Kenya	KM199302	KM199395	KM199502
*P. kenyana*	LC6633	*Camellia sinensis*	China	KX895027	KX895360	KX895246
*P. knightiae*	CBS 111963	*Knightia* sp.	New Zealand	KM199311	KM199406	KM199495
*P. knightiae*	CBS 114138 *	*Knightia* sp.	New Zealand	KM199310	KM199408	KM199497
*P. leucadendri*	CBS 121417 *	*Leucadendron* sp.	South Africa	MH553987	MH554654	MH554412
*P. licualicola*	HGUP 4057 *	*Licuala grandis*	China	KC492509	KC481683	KC481684
*P. linearis*	MFLUCC 12-0271 *	*Trachelospermum* sp.	China	JX398992	JX399027	JX399058
*P. longiappendiculata*	LC3013 *	*Camellia sinensis*	China	KX894939	KX895271	KX895156
*P. lushanensis*	LC4344 *	*Camellia* sp.	China	KX895005	KX895337	KX895223
*P. lushanensis*	LC8182	*Camellia* sp.	China	KY464136	KY464156	KY464146
*P. lushanensis*	LC8183	*Camellia* sp.	China	KY464137	KY464157	KY464147
*P. macadamiae*	BRIP 63738b *	*Macadamia integrifolia*	Australia	KX186588	KX186680	KX186621
*P. macadamiae*	BRIP 63739b	*Macadamia integrifolia*	Australia	KX186587	KX186679	KX186620
*P. macadamiae*	BRIP63739a	*Macadamia integrifolia*	Australia	KX186589	KX186681	KX186622
*P. malayana*	CBS 102220 *	*Macaranga triloba*	Malaysia	KM199306	KM199411	KM199482
*P. monochaeta*	CBS 144.97 *	*Quercus robur*	Netherlands	KM199327	KM199386	KM199479
*P. monochaeta*	CBS 440.83	*Taxus baccata*	Netherlands	KM199329	KM199387	KM199480
*P.nanjingensis*	CSUFTCC16 *	*Camellia oleifera*	China	OK493602	OK562377	OK507972
*P.nanjingensis*	CSUFTCC20	*Camellia oleifera*	China	OK493603	OK562378	OK507973
*P.nanjingensis*	CSUFTCC04	*Camellia oleifera*	China	OK493604	OK562379	OK507974
*P. nanningensis*	CSUFTCC10 *	*Camellia oleifera*	China	OK493596	OK562371	OK507966
*P. nanningensis*	CSUFTCC11	*Camellia oleifera*	China	OK493597	OK562372	OK507967
*P. nanningensis*	CSUFTCC12	*Camellia oleifera*	China	OK493598	OK562373	OK507968
*P. neolitseae*	NTUCC 17-011 *	*Neolitsea villosa*	Taiwan	MH809383	MH809387	MH809391
*P. novaehollandiae*	CBS 130973 *	*Banksia grandis*	Australia	KM199337	KM199425	KM199511
*P. oryzae*	CBS 111522	*Telopea* sp.	USA	KM199294	KM199394	KM199493
*P. oryzae*	CBS 171.26	NA	Italy	KM199304	KM199397	KM199494
*P. oryzae*	CBS 353.69 *	*Oryza sativa*	Denmark	KM199299	KM199398	KM199496
*P. pandanicola*	MFLUCC 16-0255 *	*Pandanus* sp.	Thailand	MH388361	MH412723	MH388396
*P. papuana*	CBS 331.96 *	Coastal soil	Papua New Guinea	KM199321	KM199413	KM199491
*P. papuana*	CBS 887.96	*Cocos nucifera*	Papua New Guinea	KM199318	KM199415	KM199492
*P. pallidotheae*	MAFF 240993	*Pieris japonica*	Japan	NR111022	LC311584	LC311585
*P. parva*	CBS 265.37 *	*Delonix regia*	NA	KM199312	KM199404	KM199508
*P. parva*	CBS 278.35	*Leucothoe fontanesiana*	NA	KM199313	KM199405	KM199509
*P. photinicola*	GZCC 16-0028 *	*Photinia serrulata*	China	KY092404	KY047663	KY047662
*P. portugalica*	CBS 393.48 *	NA	Portugal	KM199335	KM199422	KM199510
*P. portugalica*	LC4324	*Camellia chekiangoleosa*	China	KX895001	KX895333	KX895219
*P. pini*	MEAN 1092 *	*Pinus pinea*	Portugal	MT374680	MT374705	MT374693
*P. pinicola*	KUMCC 19-0183 *	*Pinus armandii*	China	MN412636	MN417507	MN417509
*P. rhododendri*	IFRDCC 2399 *	*Rhododendron sinogrande*	China	KC537804	KC537818	KC537811
*P. rhodomyrtus*	HGUP4230 *	*Rhodomyrtus tomentosa*	China	KF412648	KF412642	KF412645
*P. rhodomyrtus*	LC4458	*Camellia sinensis*	China	KX895010	KX895342	KX895228
*P. rhizophorae*	MFLUCC 17-0416 *	*Rhizophora apiculata*	Thailand	MK764283	MK764349	MK764327
*P. rosea*	MFLUCC 12-0258 *	*Pinus* sp.	China	JX399005	JX399036	JX399069
*P. scoparia*	CBS 176.25 *	*Chamaecyparis* sp.	NA	KM199330	KM199393	KM199478
*P. sequoiae*	MFLUCC 13-0399 *	*Sequoia sempervirens*	Italy	KX572339	NA	NA
*P. spathulata*	CBS 356.86 *	*Gevuina avellana*	Chile	KM199338	KM199423	KM199513
*P.spathuli-* *appendiculata*	CBS 144035 *	*Phoenix canariensis*	Australia	MH554172	MH554845	MH554607
*P. telopeae*	CBS 114137	*Protea* sp.	Australia	KM199301	KM199469	KM199559
*P. telopeae*	CBS 114161 *	*Telopea* sp.	Australia	KM199296	KM199403	KM199500
*P. telopeae*	CBS 113606	*Telopea* sp.	Australia	KM199295	KM199402	KM199498
*P. terricola*	CBS 141.69 *	Soil	Pacific Islands	MH554004	MH554680	MH554438
*P. thailandica*	MFLUCC 17-1616 *	*Rhizophora apiculata*	Thailand	MK764285	MK764351	MK764329
*P. trachicarpicola*	IFRDCC 2403	*Podocarpus macrophyllus*	China	KC537809	KC537823	KC537816
*P. trachicarpicola*	LC4523	*Camellia sinensis*	China	KX895011	KX895344	KX895230
*P. trachicarpicola*	MFLUCC 12-0264	*Chrysophyllum* sp.	China	JX399004	JX399035	JX399068
*P. trachicarpicola*	OP068 *	*Trachycarpus fortunei*	China	JQ845947	JQ845945	JQ845946
*P. unicolor*	MFLUCC 12-0276 *	*Rhododendron* sp.	China	JX398999	JX399030	NA
*P. unicolor*	MFLUCC 12-0275	Unidentified tree	China	JX398998	JX399029	JX399063
*P. verruculosa*	MFLUCC 12-0274 *	*Rhododendron* sp.	China	JX398996	NA	JX399061
*P. yanglingensis*	LC4553 *	*Camellia sinensis*	China	KX895012	KX895345	KX895231
*P. yanglingensis*	LC3412	*Camellia sinensis*	China	KX894980	KX895312	KX895197
*P. yunnanensis*	HMAS 96359 *	*Podocarpus macrophyllus*	China	AY373375	NA	NA
*Pestalotiopsis chinensis*	MFLUCC 12-0273 *	*Taxus* sp.	China	JX398995	NA	NA
*Pestalotiopsis dianellae*	CBS:143421 *	*Dianella* sp.	Australia	MG386051	MG386164	NA
*Pestalotiopsis dracaenae*	HGUP4037 *	*Dracaena* sp.	Thailand	MT596515	MT598645	MT598644
*Pestalotiopsis grandis-urophylla*	E-72-02	*Eucalyptus grandis*	Brazil	KU926708	KU926716	KU926712
*Pestalotiopsis humicola*	CBS 144029	*Acacia mangun*	Malaysia	MH554128	MH554801	MH554563
*Pestalotiopsis kaki*	KNU-PT-1804 *	*Diospyros kaki*	South Korea	LC552953	LC552954	LC553555
*Pestalotiopsis krabiensis*	MFLUCC 16–0260 *	*Pandanaceae*	Thailand	MH388360	MH412722	MH388395
*Pestalotiopsis menhaiensis*	YN3A1 *	*Camellia sinensis*	China	KU252272	KU252488	KU252401
*Pestalotiopsis shoreae*	ICMP: 20195	*Shorea obtusa*	Thailand	KJ503811	KJ503814	KJ503817
*Pestalotiopsis* sp.	SC3A21	*Camellia sinensis*	China	KX146689	KX146807	KX146748
*Neopestalotiopsis magna*	MFLUCC 12-0652 *	NA	France	KF582795	KF582793	KF582791
*N. saprophytica*	MFLUCC 12-0282 *	Dead plant material	China	JX398982	JX399017	JX399048
*Pseudopestolotiopsis indica*	CBS 459.78 *	*Rosa sinensis*	India	KM199381	KM199470	KM199560
*P. cocos*	CBS 272.29 *	NA	Indonesia	KM199378	KM199467	KM199553
*P. theae*	MFLUCC 12-0055 *	*Camellia sinensis*	Thailand	NR111716	JQ683711	JQ683743

**Table 3:** Newly generated sequences in this study are indicated in bold. * indicates type strains.

Species	Isolate	Substrate/Host	Country	LSU	ITS	*tub2*
*Seimatosporium pseudorosarum*	MFLUCC 14-0466 *	*Rosa canina*	Italy	NA	KT284775	NA
*Se. lichenicola*	CBS 354.90	*Fagus sylvatica*	Germany	MH554252	MH554035	MH554711
*Se. lichenicola*	MFLUCC 14-0052	*Rosa canina*	Italy	KT005514	KT005515	NA
*Se. vaccinii*	ICMP 7003	*Vaccinium ashei Reade*	New Zealand	AF382374	NA	NA
*Se. sorbi*	MFLUCC T20-1103 *	*Rocaecae*	Uzbekistan	OK642226	NA	NA
*Se. glandigenum*	AB593735	*Fagus sylvatica*	Japan	AB593735	NA	NA
*Se. lichenicola*	NBRC 32625	*Rosa canina*	United Kingdom	MH883646	MH883643	MH883645
*Se. cornii*	MFLUCC 14-0467 *	*Cornus* sp.	Italy	NG_059570	NR_156597	NA
*Se. cornii*	MFLUCC 14-1208 *	*Cornus sanguinea*	Italy	KT868531	KT868532	NA
*Se. parasiticum*	NBRC 32682	*Physocarpus amurensis*	Japan	AB593741	AB594808	NA
*Se. physocarpi*	MFLUCC 14-0625 *	*Physocarpus opulifolius*	Russia	KT198723	KT198722	MH554676
*Se. vitis*	MFLUCC 14-0051 *	*Vitis vinifera*	Italy	KR920362	KR920363	NA
*Se. pistaciae*	CBS 138865 *	*Pistacia vera*	Iran	MH878632	KP004463	MH554674
*Se. rosae*	MFLUCC 14-0621 *	*Rosa* sp.	Russia	NA	LT853105	LT853253
*Se. eucalypti*	CPC 156	*Eucalyptus smithii*	South Africa	JN871209	JN871200	NA
*Se. eucalypti*	CPC 159	*Eucalyptus smithii*	South Africa	JN871212	JN871202	NA
*Se. eucalypti*	CPC 157	*Eucalyptus smithii*	South Africa	JN871210	JN871201	NA
*Se. falcatum*	CPC 13578	*Eucalyptus alligatrix*	Australia	JN871213	JN871204	NA
*Se. obtusum*	CPC 12935	*Corymbia henryi*	Australia	JN871215	JN871206	NA
*Se. elegans*	NBRC 32674	*Melaleuca ericifolia*	Japan	AB593733	AB594801	NA
*Se. biseptatum*	CPC 13584	*Eucalyptus cypellocarpa*	Australia	JN871208	JN871199	NA
*Se. hakeae*	AB593736	*Pteridium aquilinum*	Japan	AB593736	NA	NA
*Se. grevilleae*	ICMP 10981	*Protea* sp.	South Africa	AF382372	NA	NA
*Se. leptospermi*	ICMP 11845	*Leptospermum scoparium*	New Zealand	AF382373	NA	NA
*Se. mariae*	NBRC 32681	*Correa reflexa*	Japan	AB593740	AB594807	NA
*Se. walkeri*	CPC 17644	*Eucalyptus* sp.	Australia	JN871216	JN871207	NA
*Se. rhombisporum*	MFLUCC 15-0543 *	*Vaccinium myrtillus*	Italy	NG_059566	NA	NA
*Sporocadus cornicola*	MFLUCC 14-0448 *	*Cornus sanguinea*	Italy	NA	KU974967	NA
*Sp. cornicola*	CBS 143889	*Cornus sanguinea*	Germany	MH554326	MH554121	MH554794
*Discostroma stoneae*	NBRC 32690	NA	Japan	AB593729	AB594797	NA
*Sarcostroma restionis*	CBS 118154	Culm litter of *Restio filiformis*	South Africa	DQ278924	DQ278922	MH554651
*Sar. restionis*	CBS 118153	Culm litter of *Restio filiformis*	South Africa	DQ278925	DQ278923	MH554650
*Sar. bisetulatum*	CBS 122695	*Protea acaulos*	South Africa	NA	EU552155	NA
*Strickeria kochii*	C138	*Robinia pseudoacacia*	Austria	NA	KT949917	NA
*Str. kochii*	C143	*Robinia pseudoacacia*	Austria	NA	KT949918	NA
*Discosia neofraxinea*	MFLU 15-0375 *	*Fagus sylvatica*	Italy	KR072672	KR072673	NA
*Dis. fraxinea*	NTIT469	Dead leaf	Italy	KF827439	KF827435	KF827472
*Dis. brasiliensis*	NTCL097-2	Dead leaf	Thailand	KF827438	KF827434	KF827471
*Dis. brasiliensis*	NTCL095	Dead leaf	Thailand	KF827437	KF827433	KF827470
*Dis. brasiliensis*	NTCL094-2	Dead leaf	Thailand	KF827436	KF827432	KF827469
*Dis. pini*	MAFF 410149	*Pinus densiflora*	Japan	AB593708	AB594776	AB594174
*Dis. celtidis*	MFLU 18-2581	*Celtis formosana*	Taiwan	MW114406	MW114327	NA
*Dis. rubi*	CBS 143893	*Rubus phoenicolasius*	USA	MH554334	MH554131	MH554804
*Dis. pseudoartocreas*	CPC 21117	*Tilia* sp.	Austria	KF777214	KF777161	NA
*Dis. pseudoartocreas*	CBS 136438	NA	Austria	MH877640	MH866098	MH554672
*Dis. aff. artocreas*	NBRC 8975	*Poa pratensis*	Japan	AB593705	AB594773	AB594172
*Dis. querci*	MFLUCC 16-0642	*Quercus* sp.	United Kingdom	MG815830	MG815829	NA
*Discosia* sp.	KT2188	Unknown leaves	Japan	AB593713	AB594781	AB594179
*Discosia* sp.	KT2192	Unknown leaves	Japan	AB593714	AB594782	AB594180
*Dis. italica*	MFLU 14-0298C	*Fagus sylvatica*	Italy	KM678044	KM678041	NA
*Dis. italica*	MFLU 14-0298B	*Fagus sylvatica*	Italy	KM678046	KM678043	NA
*Dis. fagi*	MFLU 14-0299A	*Fagus sylvatica*	Italy	KM678048	KM678040	NA
*Dis. fagi*	MFLU 14-0299B	*Fagus sylvatica*	Italy	KM678047	KM678039	NA
*Dis. fici*	MFLU 19-2704	*Ficus septica*	Taiwan	MW114409	MW114330	NA
*Dis. macrozamiae*	CPC 32113	*Macrozamia miquelii*	Australia	MH327855	MH327819	MH327894
*Adisciso yakushimensis*	MAFF 242774 *	*Symplocos prunifolia*	Japan	AB593721	AB594789	AB594187
*Adi. tricellularis*	NBRC 32705	*Rhododendron indicum*	Japan	AB593728	AB594796	AB594188
*Immersidiscosia eucalypti*	KT2091	*Quercus myrsinifolia*	Japan	AB593722	AB594790	NA
*Imm. eucalypti*	KT2191	Unknown leaves	Japan	AB593725	AB594793	NA
*Imm. eucalypti*	KT2115	*Quercus myrsinifolia*	Japan	AB593723	AB594791	NA
*Ciliochorella mangiferae*	MFLUCC 12-0310	Dead leaf	Thailand	KF827445	KF827444	KF827478
*C. castaneae*	HHUF:28800	*Fraxinus lanuginosa, Viburnum dilatatum, Cercidiphyllum japonicum*, *Kalopanax pictus*, *Fagus crenata, Betula ermanii*, *Pteridium aquilinum*	Japan	AB433278	NA	NA
*C. castaneae*	HHUF:28799	*Fraxinus lanuginosa, Viburnum dilatatum, Cercidiphyllum japonicum, Kalopanax pictus, Fagus crenata* and *Betula ermanii*, as well as on a fern, *Pteridium aquilinum*	Japan	AB433277	NA	NA
*C. phanericola*	MFLUCC 14-0984	Dead leaves of *Phanera purpurea*	Thailand	KX789681	KX789680	KX789682
***C. dipterocarpi***	**MFLUCC 22-0132**	***Dipterocarpus alatus***	**Thailand**	**OP912990**	**OP912991**	**OQ127637**
***C. dipterocarpi***	**MFLUCC 23-0023**	***Dipterocarpus alatus***	**Thailand**	**OQ657981**	**OQ657982**	**OQ657298**
*P. versicolor*	BRIP 14534	*Psidium guajava*	Australia	AF382357	AF405298	NA
*P. parva*	CBS 278.35	*Leucothoe fontanesiana*	NA	KM116205	KM199313	KM199405

**Table 4:** Newly generated sequences in this study are indicated in bold. * indicate type strains.

Species	Isolate	Host/Substrate	Country	LSU	ITS	*rpb2*	*tef1-a*
*Dis. tricellularis*	NBRC 32705	*Rhododendron indicum*	Japan	NG_042334	NR_119411	NA	NA
*Dis. yakushimensis*	HHUF 29671 *	*Symplocos prunifolia*	Japan	AB593721	AB594789	NA	NA
*Bartalinia robillardoides*	CBS 122705 *	*Callistemon speciosus*	Italy	AF382366	AF405301	NA	NA
*Bar. rosicola*	MFLUCC 17-0645 *	*Rosa canina*	Italy	MG828988	MG828872	NA	NA
*Broomella rosae*	MFLU 16-0244	*Rosa canina*	Italy	MG828990	MG828874	NA	NA
*Bro. vitalbae*	MFLUCC 15-0023	*Clematis vitalba*	Italy	KP757751	KP757755	NA	KP757763
*Dis. artocreas*	NBRC 8975	*Poa pratensis*	Japan	AB593705	AB594773	NA	NA
*Dis. pini*	MAFF 410149	*Pinus densiflora*	Japan	AB593708	AB594776	NA	NA
*Hyalotiella spartii*	MFLUCC 15-0024	*Spartium junceum*	Italy	KP757753	KP757757	NA	NA
*Hymenopleella hippophaeicola*	CBS 140410 *	*Hippophaë rhamnoides*	Austria	NG_064296	NR_154078	MH554919	MH554436
*Imm. eucalypti*	MFLU 16-1372	*Quercus* sp.	Italy	AB593722	AB594790	NA	NA
*Monochaetia kansensis*	PSHI2004Endo1030	*Rhododendron* sp.	India	DQ534035	DQ534044	NA	NA
*Mon. monochaeta*	CBS 191.82			AF382370		NA	NA
*Morinia acaciae*	CBS 137994 *	*Prunus salicinacv. ‘Omega’*	New Zealand	MH554221	MH554002	MH554914	MH554431
*Mor. pestalozzioides*	F-090354	*Sedum sediforme*	Spain	NA	AY929325	NA	AY929314
*Neopestalotiopsis protearum*	CBS 114178 *	*Leucospermum cunciforme*	Zimbabwe	NA	LT853103	MH554873	KM199542
*Neo. rosae*	CBS 101057 *	*Rosae* sp.	New Zealand	KM116245	KM199359	MH554850	KM199523
*P. knightiae*	CBS 114138 *	*Knightia* sp.	New Zealand	KM116227	KM199310	MH554870	KM199497
*P. malayana*	CBS 102220 *	*Macaranga triloba*	Malaysia	KM116238	KM199306	NA	KM199482
*Pseudopestalotiopsis cocos*	CBS 272.29 *	*Cocos nucifera*	Indonesia	KM116276	KM199378	MH554938	KM199553
*Pse. theae*	MFLUCC 12-0055 *	*Camellia sinensis*	Thailand	NA	NR_111716	NA	NA
*Robillarda africana*	CBS 122.75 *	NA	South Africa	KR873281	KR873253	MH554896	MH554414
*Rob. australiana*	CBS 143882 *	NA	Australia	MH554301	MH554091	MH555005	MH554525
***Rob. australiana***	**MFLUCC 22-0121**	***Dipterocarpus alatus***	**Thailand**	**OP906280**	**OP906277**	**OQ127641**	**OQ127640**
*Rob. mangiferae*	KUMCC 18-0180 *	*Mangifera*	Yunnan, China	MK353086	MK353084	NA	NA
*Rob. roystoneae*	CBS 115445 *	*Roystonea regia*	Hong Kong	KR873282	KR873254	MH554880	KR873310
*Rob. sessilis*	CBS 101440	*Heterodera glycines*	USA	NA	KR873255	NA	KR873311
*Rob. sohagensis*	CBS H–23861	*Phoenix dactylifera*	Egypt	NA		NA	NA
*Robillarda* sp.	CPC 25020	NA	NA	KR873287	KR873259	NA	KR873315
*Robillarda* sp.	MS9788	NA	NA	LC309276		NA	NA
*Rob. terrae*	CBS 587.71 *	Soil	India	KJ710459	KJ710484	MH554971	MH554493
*Rob. aquatica*	MFLUCC 21–0217	Decaying wood submerged in freshwater	Thailand	NA	OL504777	NA	NA
*Se. rosae*	MFLUCC 14-0621 *	*Rosa* sp.	Russia	NA	LT853105	LT853153	LT853203
*Sporocadus rosigena*	MFLU 16-0239 *	*Rosa canina*	Italy	MG829069	MG828958	NA	NA
*Se. rosicola*	MFLUCC 15-0564	*Rosa canina*	Italy	MG829070	MG828959	NA	NA
*Seiridium marginatum*	CBS 140404 *	*Rosa canina*	Austria	MH878681	NA	NA	NA
*Seir. phylicae*	CPC 19965	*Phylica arborea*	Tristan da Cunha	KC005809	KC005787	LT853141	LT853190
*Seri. marginatum*	CBS 140403	*Rosa canina*	France	NG_064297	NR_156602	LT853149	LT853199
*Seri. marginatum*	SEI	*Rosa canina*	France	NA	KT949915	NA	NA
*Strickeria kochii*	C138	*Robinia pseudoacacia*	Austria	NA	KT949917	NA	NA
*Stri. kochii*	CBS 140411 *		Austria	NG_064298	NR_154423	MH554920	MH554437
*Truncatella hartigii*	CBS 118148	*Rhodocoma capensis*	South Africa	DQ278928	DQ278913	MH554888	MH554406
*Tru. restionacearum*	CMW 18755	*Restio filiformis*	South Africa	DQ278929	DQ278915	NA	NA
*Phlogicylindrium eucalyptorum*	CBS 111689	*Eucalyptus nitens*	Australia	KF251708	KF251205	KF252210	KF253161
*Phlo. uniforme*	CBS 131312 *	*Eucalyptus cypellocarpa*	Australia	JQ044445	JQ044426	MH554910	MH704609

**Table 5:** The nucleotide evolutionary models calculated for Bayesian inference.

Taxa	Evolutionary Model				
	ITS	LSU	*tub2*	*tef1-a*	*rpb2*
*Pestalotiopsis*	TIM3 + I + G		HKY + I + G	TIM2 + I + G	
*Ciliochorella*	SYM + I + G	TIM2 + I + G	HKY + I + G		
*Robillarda*	TPM3uf + I + G	TIM2 + I + G		TIM2 + G	TIM2 + G

## Data Availability

All sequences generated in this study were submitted to GenBank (https://www.ncbi.nlm.nih.gov (accessed on 4 April 2023)).
